# Ultrafast Synthesis of Urchin-Like Rutile TiO_2_ by Single-Step Microwave-Assisted Method

**DOI:** 10.3390/nano8080630

**Published:** 2018-08-20

**Authors:** Liqin Xiang, Yuchi Liu, Yang Liu, Chen Zheng, Xiaopeng Zhao

**Affiliations:** Smart Materials Laboratory, Department of Applied Physics, Northwestern Polytechnical University, Xi’an 710072, China; 2017262331@mail.nwpu.edu.cn (Y.L.); daguoyliu@mail.nwpu.edu.cn (Y.L.); zhengchen2603@mail.nwpu.edu.cn (C.Z.)

**Keywords:** microwave synthesis, rutile TiO_2_, urchin-like, ultrafast, one-step

## Abstract

The preparations of crystal titanium dioxide (TiO_2_) are often time-consuming multistep processes involving high temperature. Rapid and efficient methods to obtain TiO_2_ with anatase or rutile phase are desirable. In this paper, we describe an ultrafast single-step method to obtain urchin-like rutile TiO_2_ particles via microwave irradiation. In the procedure, TiCl_4_ aqueous solution was used as a reactant and toluene was used as a solvent. It takes only a few minutes without any further heat treatment. The samples were characterized by scanning electron microscopy (SEM), transmission electron microscopy (TEM), X-ray diffraction (XRD), and thermal gravimetric analysis (TGA). The effect of temperature, irradiation time and the ratio of precursor to solvent on the morphology and crystal structure were studied. The results show urchin-like rutile TiO_2_ with high stability is formed after only 5 min microwave irradiation at 135 °C.

## 1. Introduction

As one of most important semiconductors, titanium dioxide (TiO_2_) is very popular in many fields due to its outstanding properties [1,2,3]. TiO_2_ and its composites have been widely used as photocatalyst, solar cell, antibacterial agent, and so on [4,5,6]. In last ten years, TiO_2_ with multilevel interior structure such as urchin-like TiO_2_ microspheres has attracted much attention [7,8,9]. The unique microstructure endows particles with the advantages of nanomateials and micromaterials [8,9]. Especially, urchin-like TiO_2_ shows outstanding photocatalysis ability because more incident lights can be absorbed through multiple-reflection of the urchin-like hierarchical microstructure. Furthermore, compared with nanoparticles, it is easier to separate the urchin-like particles from waste-water by filtration or sedimentation method after photocatalytic reaction [8]. Other enhanced properties have also been observed in novel applications such as visible light applications [9], solar cell [10], and enhanced Visible-Light-Responsive H_2_ Production [11].

The TiO_2_ particles with different morphologies have been successfully prepared by various techniques [12,13,14]. Conventional methods including sol-gel, hydrothermal, and solvothermal processes are usually adopted to prepare TiO_2_ materials [15]. They generally involves several steps, long reaction time or high temperature to obtain crystalline TiO_2_ by conventional methods [15]. Hydrothermal or solvothermal methods are usually adopted to prepare urchin-like TiO_2_ particles. In the previous work, our group had prepared rutile TiO_2_ urchin-like spheres by the solvothermal method, which took at least 16 h [8]. Although the electrochemistry method can reduce the reaction time of urchin-like TiO_2_, it still takes several tens of minutes [10]. Therefore, the synthesis of crystalline TiO_2_ via a rapid, reproducible, and simple method is a strong challenge and is desired.

The microwave-assisted method is distinguished for short reaction time and high energy efficiency [16]. A lot of materials have been successfully prepared by microwave-assisted method [17,18,19,20]. Here, we present a facile microwave-assisted solvothermal process to obtain the rutile TiO_2_ particles with urchin-like morphology in just a few minutes without any further heat treatment. All the reactions were easy to finish in a microwave quartz tube. The effect of temperature, irradiation time and the ratio of reactants to solvent on the morphology and the crystal structure of the TiO_2_ particles were investigated systematically.

## 2. Experimental Section

### 2.1. Synthesis

The TiO_2_ particles were prepared by a microwave-assisted solvothermal reaction. All the chemicals were of analytical grade and were used as obtained without further purification. The chemicals titanium tetrachloride (TiCl_4_), anhydrous ethanol, and toluene were purchased from Kermel Chemical Reagent Co. Ltd (Shanghai, China). Deionized water was used throughout the syntheses. Titanium tetrachloride (TiCl_4_) was dissolved into distilled water in an ice-water bath under vigorous stirring to obtain a 40 wt% TiCl_4_ aqueous solution. In a typical experiment, 1 mL of TiCl_4_ aqueous solution was added dropwise into 15 mL toluene in a quartz tube under mild stirring for 30 min. The quartz tube with mixture was loaded into a mono-mode microwave synthesis system (CEM explorer). The microwave system was operated at a frequency of 2.45 GHz and power of 150–250 W, the sample temperature was ramped to 135 °C with 20 °C/min and kept at the temperature for 1 to 30 min. The precipitate was separated by centrifugation, washed with ethanol, and dried at 70 °C.

### 2.2. Characterization 

The morphology was observed by scanning electron microscopy (SEM, JSM-6700, JEOL Ltd., Tokyo, Japan) and transmission electron microscopy (TEM, JEOL-3010, JEOL Ltd., Tokyo, Japan). The crystal structure was characterized by powder X-ray diffraction (XRD, Philips X’Pert Pro, the Netherland) with CuK_α_ irradiation (40 kV/35 mA) and step size of 0.033° in the 2θ range of 10–90°. The thermal gravimetric analysis of samples was determined by the thermogravimetric analyzer (TGA, Netzsch STA449F3, Netzsch, Bavaria, Germany) with a heating rate of 10 °C/min within 35–800 °C under air atmosphere. 

### 2.3. Photocatalytic Measrement 

Analytical grade methyl blue dye (MB, Tianjin Chemical Reagent Co. Ltd., Tianjin, China) was served as the target organic pollutant for photocatalytic experiments. The typical photocatalytic test was performed at 25 °C. First, 30 mg photocatalyst was added into 30 mL MB aqueous solution (40 mg/L). The solution was stirred in darkness for 3 h to achieve the equilibrium absorption of MB. Then, the suspension was exposed to UV-Vis light irradiation using a 20 W low pressure mercury lamp which has spectral energy distribution centered at *λ* = 365, 405, 436, 547, and 578 nm. After a regular interval, 2 mL of suspension was taken from the reactor. Finally, the catalyst was separated by centrifugation and the MB solution was analyzed by UV/V spectrophotometer (U-4100, HITACHI, Tokyo, Japan). The change of normalized temporal concentration (*C*/*C*_0_) of MB during photodegradation was compared to evaluate the photocatalytic efficiency. Here, the *C*/*C*_0_ is proportional to the normalized maximum absorbance (*A*/*A*_0_) and derives from the change in the dye’s absorption peak (*λ* = 590 nm).

## 3. Results and Discussion

**Figure 1:**
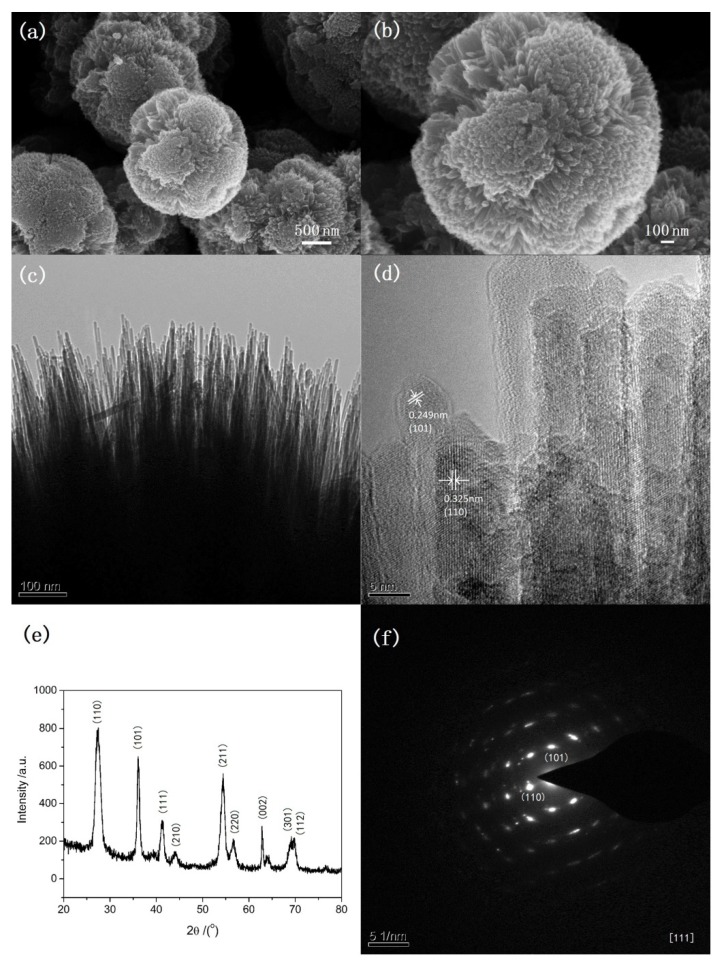
Scanning electron microscope (SEM) images (**a**,**b**), transmission electron microscope (TEM) images (**c**,**d**), X-ray diffraction (XRD) (**e**) and selected area electron diffraction (SAED) pattern (**f**) of the urchin-like titanium oxide (TiO_2_) synthesized at 135 °C under microwave irradiation for 5 min.

The SEM images, TEM images, XRD and selected area electron diffraction (SAED) patterns of the typical sample are shown in Figure 1. This sample was prepared at 135 °C under microwave irradiation for 5 min. 1 mL of TiCl_4_ aqueous solution (40 wt%) is used as the only reactant and 15 mL toluene is used as solvent. There is no any surfactant in the procedure. SEM images (Figure 1a,b) show that the particles are urchin-like spheres with diameters about 2–3 μm. TEM images (Figure 1c,d) show that nanoneedles with diameters about 5–10 nm assemble radially on the surface of spheres. The outlines of nanoneedles can be only identified near the surface of the microspheres. Although the morphology of TiO_2_ from this microwave-assisted method appears less urchin-like than that of the TiO_2_ from the previous solvothermal method, the crystalline purity appears of a higher quality [8]. The high-resolution TEM (HRTEM) images of tip of nanoneedles in Figure 1d and the corresponding SAED pattern in Figure 1f indicate the single crystalline nature of nanoneedles. According to the measured plane distance in HRTEM and SAED pattern, the crystal phase of the sample is a rutile structure. This can be further verified by the XRD pattern in Figure 1e. Two factors may be attributed to the fact that microwave helps to shorten the process. One is the fast and homogeneous heating from microwave irradiation, which favors the fast formation of crystalline TiO_2_. The other may be so called localized superheating by specific microwave absorption by polar components (TiO_2_) of a reaction making them more reactive under microwave irradiation when compared to thermal heating [21].

**Figure 2:**
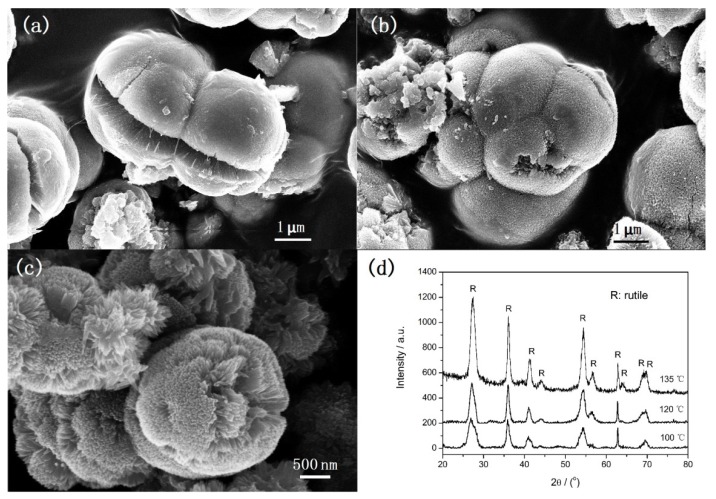
SEM images (**a**–**c**) and XRD (**d**) of TiO_2_ synthesized at various temperatures under microwave irradiation for 5 min: (**a**) 100 °C; (**b**) 120 °C; (**c**) 135 °C.

Figure 2 shows SEM images (a–c) and XRD patterns (d) of samples obtained at various reaction temperatures under microwave irradiation for 5 min. It can be observed that the morphology of samples varies with the reaction temperature. At 100 °C, microspheres with diameters about 3 μm and irregular particles with different size are formed. The surface of microspheres is rough and cracks can be observed. The corresponding XRD peaks correspond to rutile structure only but the peak intensity is very wide (Figure 2d). It indicates the rutile sample can be formed at 100 °C but the crystalline size is so small. When the temperature is increased to 120 °C, aggregates consisting of microspheres with rough surface and irregular particles are obtained (Figure 2b). The corresponding XRD peaks also correspond to rutile structure and the crystallization level is improved. When the temperature is increased to 135 °C, well-defined urchin-like microspheres are formed (Figure 2c). It is obvious that, comparing the diffraction peaks of the samples obtained at 100 °C and 120 °C, the crystallization level of the sample obtained at 135 °C is further improved. Therefore, it can be concluded that the temperature has a significant influence on the morphology and crystal size, but no influence on the crystal phase.

**Figure 3:**
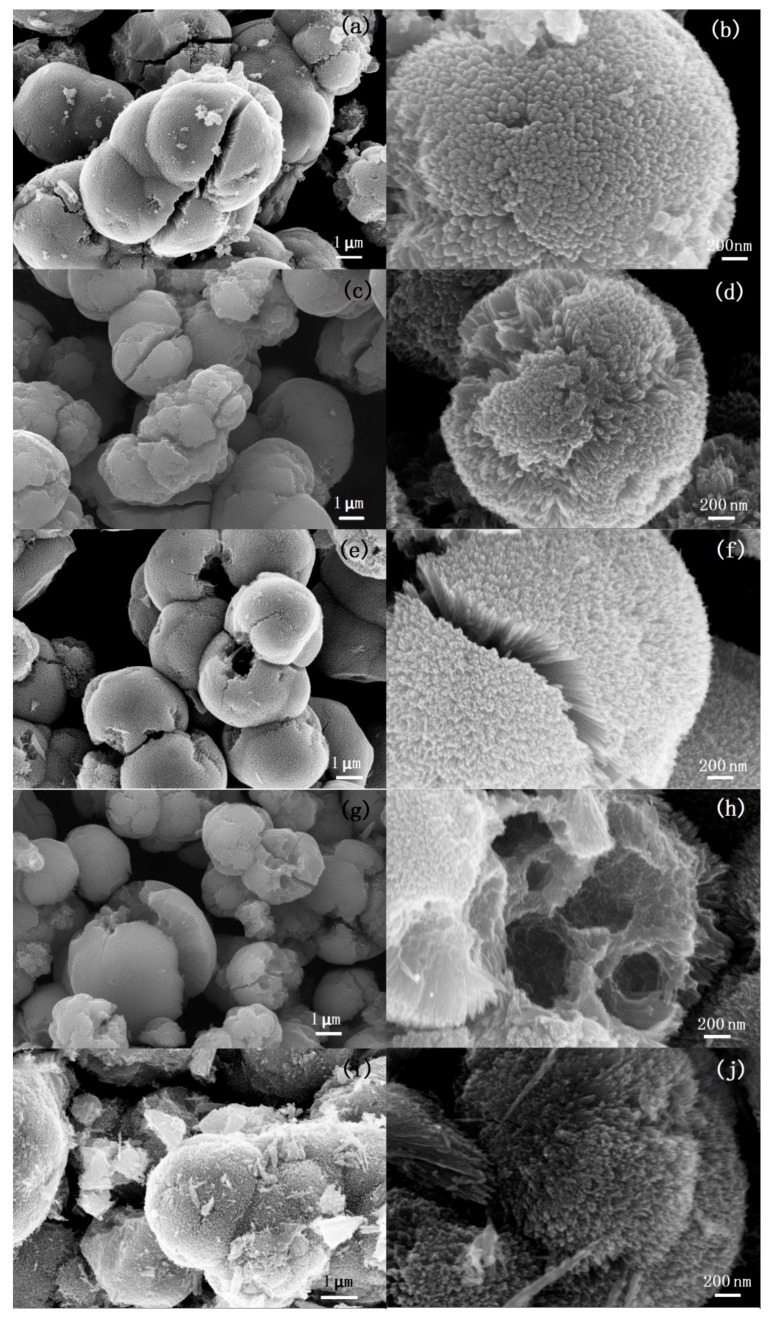
SEM images of the TiO_2_ samples synthesized at 135 °C under (**a**,**b**) 1 min, (**c**,**d**) 5 min, (**e**,**f**) 10 min, (**g**,**h**) 20 min, and (**i**,**j**) 30 min microwave irradiation.

Figure 3 shows SEM images of TiO_2_ samples synthesized at 135 °C under different microwave irradiation times. As shown in Figure 3a, the sample obtained under 1 min microwave irradiation are aggregates consisting of mirospheres with diameters of about 3–4 μm. Cracks can be observed obviously on the surface of the microspheres. There are also some irregular nanoparticles that are found in the sample. The SEM image with a higher magnification (Figure 3b) shows that the microspheres have a rough surface. There are many bumps consisting of nanoneedles on the surface of the microspheres. Figure 3c,d show the SEM images of the sample obtained after 5 min microwave irradiation. It can be found that aggregates are formed by the assembled urchin-like hierarchical TiO_2_ microspheres with a diameter of about 2 μm. There are few separated nanoparticles that could be observed. The surface of the microspheres is covered with nanoneedles assembling radially. When the microwave irradiation time is increased to 10 min, cracks and holes can be observed on the surface of some particles (Figure 3e). It can be observed from the edge of the cracks that the nanoneedles grow radially from the core of the microspheres (Figure 3f). Many crashed microspheres can be observed in the sample obtained after 20 min microwave irradiation (Figure 3g).

Furthermore, from the crashed microspheres we can find that the microspheres possess hollow or porous structures. High-resolution imaging (Figure 3h) shows that the pores in the microspheres have a broad pore size distribution ranging from 50 to 300 nm. When the microwave irradiation time is further increased to 30 min, the morphology of the samples (Figure 3i,j) changes little but many fragments of crashed microspheres are observed. Based on the time-dependent evolution of morphology, it can be found that the irradiation time plays a crucial role on the interior structure of the particles. Prolonging irradiation time leads to form hollow or porous interior structure. The urchin-like particles with solid, hollow, or porous hierarchical structure can be prepared by adjusting the irradiation time.

**Figure 4:**
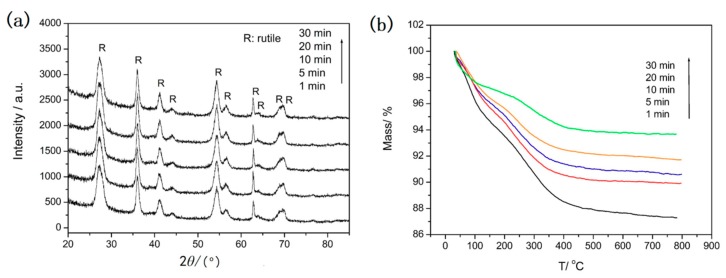
XRD patterns (**a**) and TG curves (**b**) of the TiO_2_ samples synthesized at 135 °C under different microwave irradiation time.

Figure 4 shows the corresponding XRD patterns and TGA curves of samples after different microwave irradiation times at 135 °C. The XRD patterns in Figure 4a show that only the diffraction peaks of rutile are observed and no obvious differences are found when the irradiation time changes from 1 to 30 min. The results show that a high crystal degree of rutile has formed after only 1 min microwave irradiation. This indicates that the microwave-assisted method is a very fast way to obtain rutile TiO_2_. The TG curves of the samples are showed in Figure 4b. It can be observed that the same behavior occurred for the samples in the temperature range 35–800 °C. All the samples undergo significant weight loss from 35 °C to 350 °C due to the dehydration of the physically absorbed water molecules and the removal of the residual solvents such as ethanol. The weight loss of 10.7%, 10.1%, 9.4%, 8.3%, and 5.2% is observed before 350 °C for the samples after microwave irradiation for 1 min, 5 min, 10 min, 20 min, and 30 min. The weight loss of the sample after microwave irradiation for 1 minute is about 2% and that of other samples is only about 1% throughout the temperature range of 350–800 °C. The results indicate the high stability of these samples. It is noteworthy that prolonging irradiation time only reduces the quantity of physically absorbed water and the residual solvent molecules in the sample. Combining the results of SEM, XRD with TG of the samples, it can be concluded that 5 min microwave irradiation is enough to obtain urchin-like rutile TiO_2_ with high stability.

**Figure 5:**
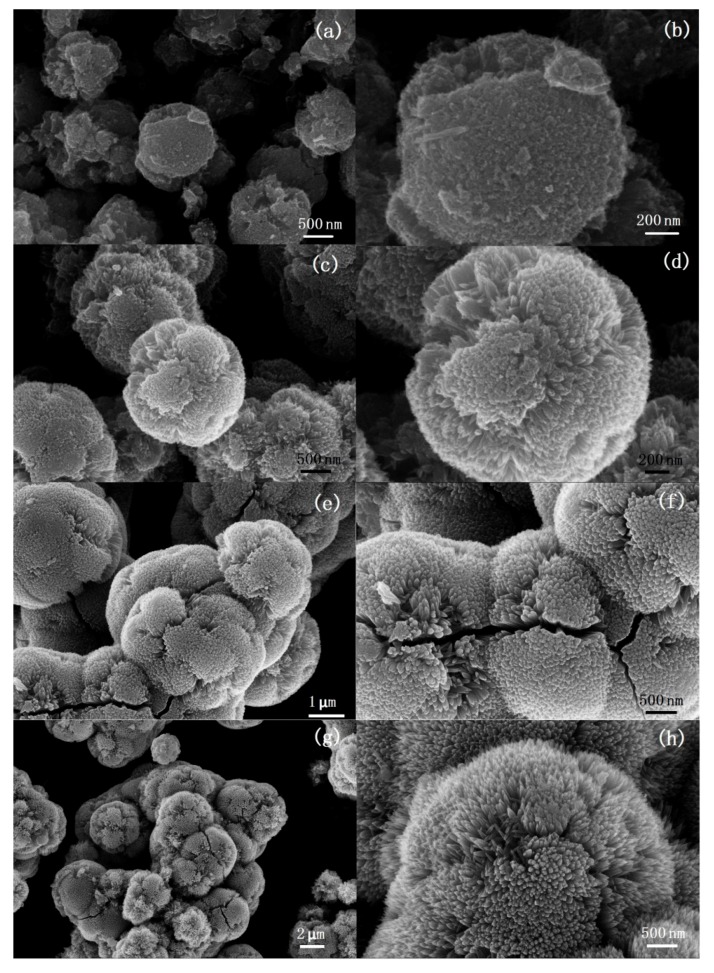
SEM images of TiO_2_ samples synthesized at the different volume ratio of TiCl_4_ solution to toluene. (**a**,**b**) 1:30; (**c**,**d**) 1:15; (**e**,**f**) 1:10; (**g**,**h**) 1:7.5.

Figure 5 shows the morphology of samples synthesized at the different volume ratio of TiCl_4_ solution to toluene when other experimental conditions, such as the concentration of TiCl_4_ solution (40 wt%), reaction temperature (135 °C), microwave irradiation time (5 min), and toluene volume (15 mL), are fixed. When the volume ratio of TiCl_4_ solution to toluene is 1:30, only irregular particles with diameters of about 2–3 μm are obtained. The surface of the particles is rough, but no urchin-like microstructure can be found (Figure 5a,b). When the ratio of TiCl_4_ to toluene is 1:15, 1:10, and 1:7.5, aggregates made of urchin-like microspheres can be observed clearly. The diameters of aggregates and the length of nanoneedles on the particle surface increases with the ratio of TiCl_4_ to toluene. The difference of morphology related to the ratio of TiCl_4_ to toluene may be attributed to the change of HCl concentration and the water content. HCl is produced in the process of decomposition of TiCl_4_. The content of water and HCl, which has been identified to be important to the morphology and crystal phase of TiO_2_, increases with the ratio of TiCl_4_ solution to toluene.

**Figure 6:**
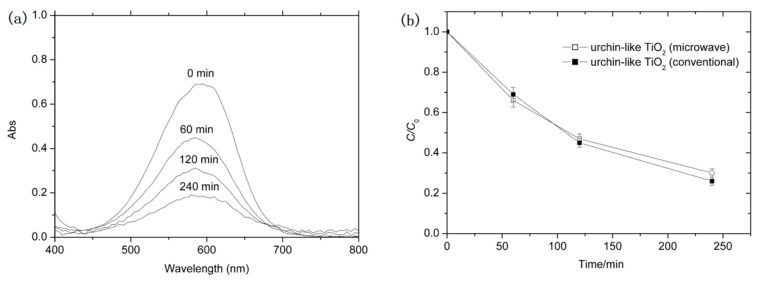
(**a**) The absorbance spectra of methyl blue as a function of irradiation time when the TiO_2_ samples synthesized at 135 °C under 5 min microwave irradiation is used as a photocatalyst; (**b**) Photodegradation of methyl blue for urchin-like TiO_2_ synthesized by the microwave-assisted method and conventional solvothermal method under UV-Vis light.

Finally, by comparing with urchin-like TiO_2_ obtained by the conventional solvothermal method, we evaluated the photocatalytic efficiency of present urchin-like TiO_2_ as a photocatalyst. Methyl blue dye was served as the target organic pollutant. Figure 6 shows the typical absorbance spectra of MB solution with irradiation time and the change of normalized temporal concentration (*C*/*C*_0_) of MB during photodegradation. It is found that the absorbing intensity of MB decreases with irradiation time, indicating the rapid photodegradation of MB. The photocatalytic degradation efficiency of urchin-like TiO_2_ obtained by microwave-assisted method is only slightly lower than that of urchin-like TiO_2_ obtained by conventional solvothermal method. This may be attributed to their similar urchin structure, crystal phase, or special surface area, etc. A more detailed evaluation about the urchin-like TiO_2_ as photocatalyst and other applications is in progress and we will report them in a future communication.

## 4. Conclusions

Urchin-like rutile TiO_2_ was quickly prepared via a one-step microwave-assisted method. TiCl_4_ solution was the only precursor and toluene was the media. The synthesis was carried out at 135 °C under microwave irradiation for only 1–30 min. All the procedures were conducted in a single vessel. No high temperature was involved in the process. The temperature, irradiation time and the ratio of precursor to solvent had an effect of on the morphology and the crystal structure. The crystallization level improves with the temperature and urchin-like particles with high level crystallization can be formed when the temperature is 135 °C. The crystal structure changes little when the irradiation time changes from 1 min to 30 min. The length of the nanoneedles on the surface increases with the ratio of TiCl_4_ solution to toluene. This study provides an ultrafast and highly efficient method for the controllable synthesis of urchin-like rutile TiO_2_.
